# Supplementary figures and images for: Increased immunogen valency improves the maturation of vaccine-elicited HIV-1 VRC01-like antibodies
Source: PLoS Pathog. 2025 May 29;21(5):e1013039. doi: 10.1371/journal.ppat.1013039 (PMC12289002; doi:10.1371/journal.ppat.1013039)

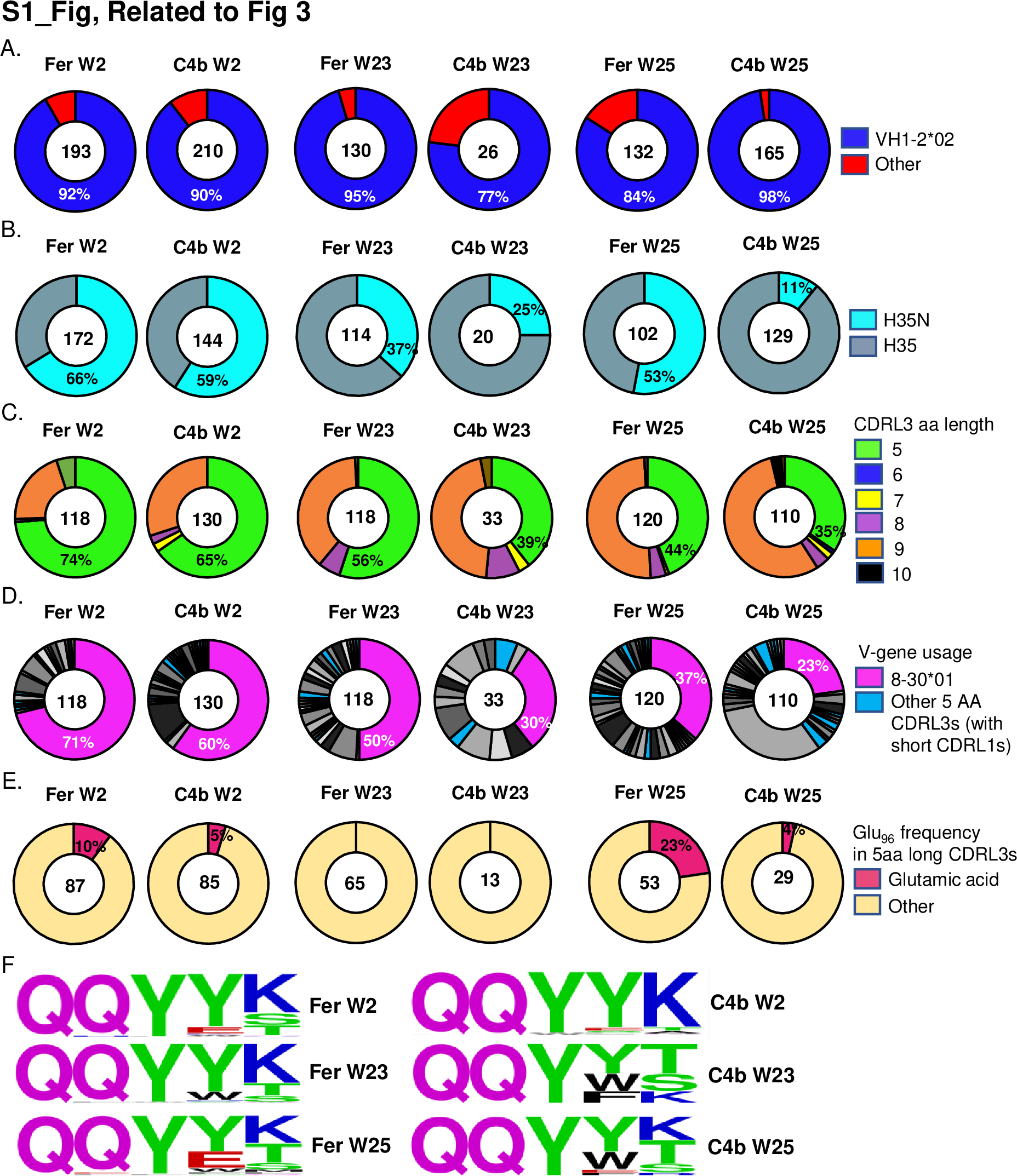

Supplement: S1 Fig — Pie charts indicate HC (A, B) and LC (C to F) characteristics from individually sorted Env-specific B cells from pooled mouse samples. The number of HC and LC sequences analyzed is shown in the middle of each pie chart. (A) VH-gene usage, (B) HCs with the H35N mutation are shown. (C) aa length of the CDRL3 domains in the LC, (D) LC-gene usage, where shades of grey/black slices represent non 5-aa long CDRL3s and blue indicates other 5-aa CDRL3s. (E) Presence of Glu96 within the LC sequences with 5-aa long CDRL3 domains, and (F) Logo plot showing CDRL3 region from the two NP groups at the indicated time points. (TIF) [file ppat.1013039.s001.tif]

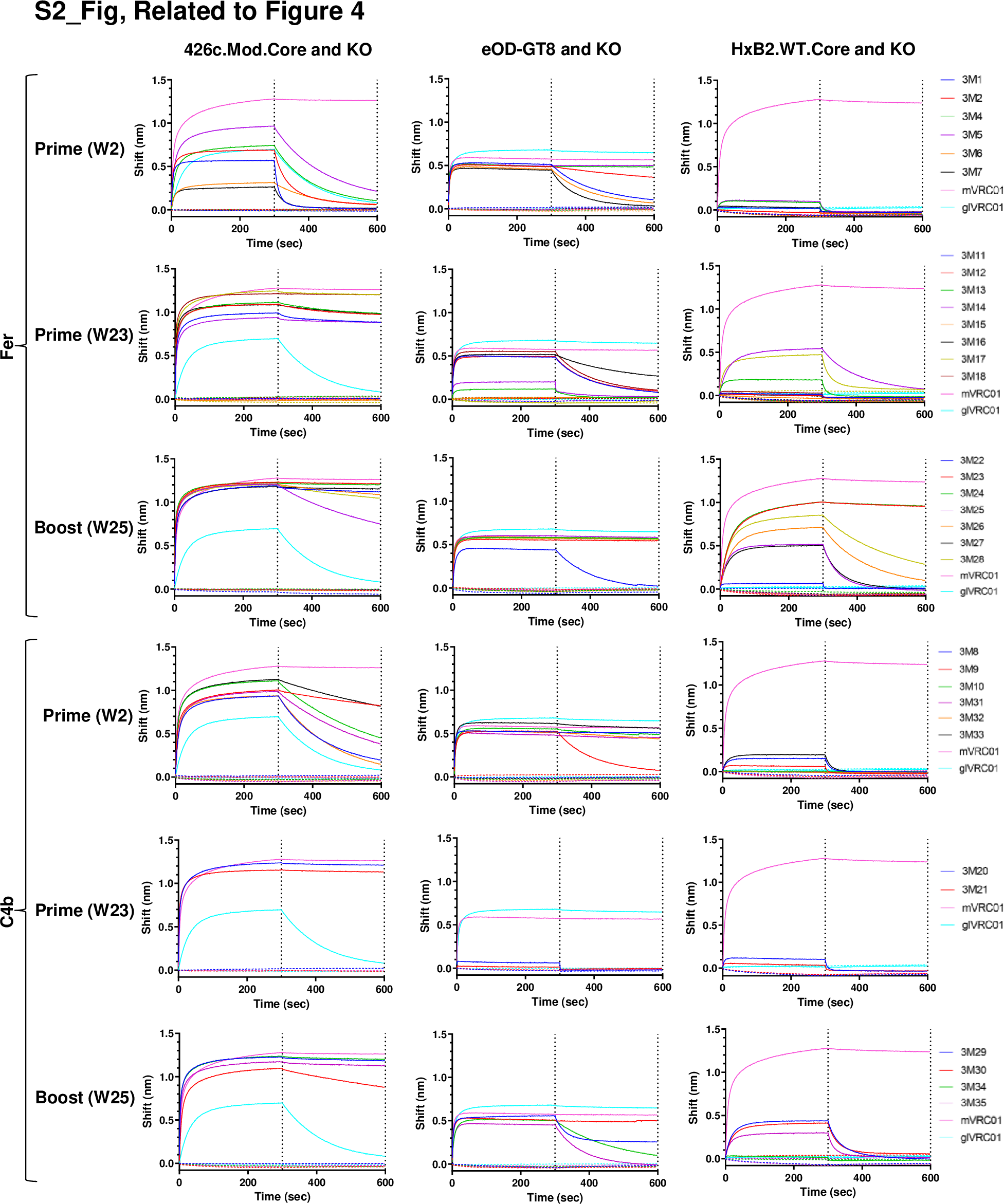

Supplement: S2 Fig — mAbs were evaluated against the indicated soluble monomeric Envs (solid lines) and their knock-outs (KO; corresponding color dotted lines) using BLI assay. mVRC01 (solid pink line) and glVRC01 (solid cyan line) were included as internal controls. Black dotted lines indicate end of association and dissociation phases. (TIF) [file ppat.1013039.s002.tif]

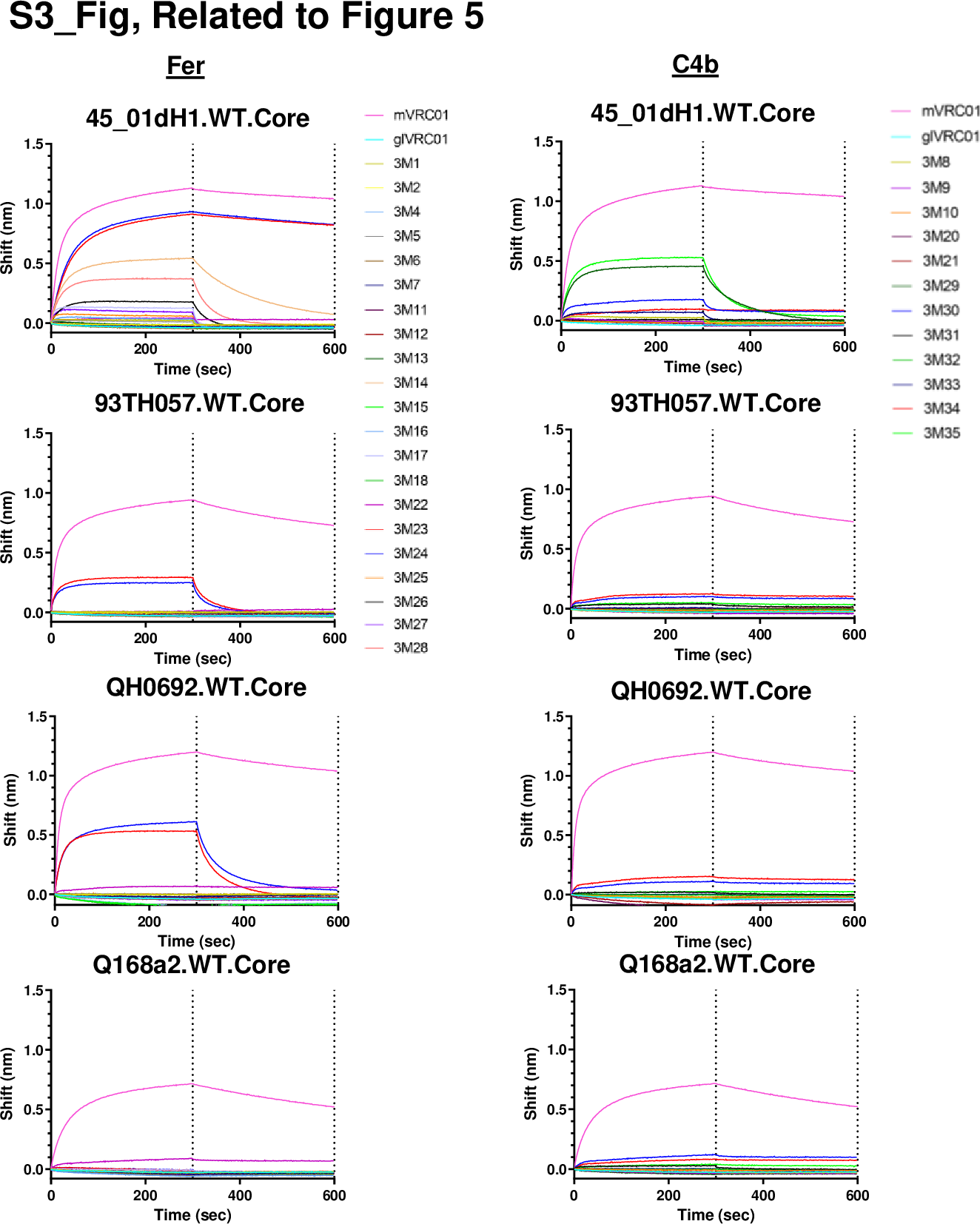

Supplement: S3 Fig — Core Envs. mVRC01 (solid pink line) and glVRC01 (solid cyan line) were included as internal controls. Black dotted lines indicate end of association and dissociation phases. (TIF) [file ppat.1013039.s003.tif]

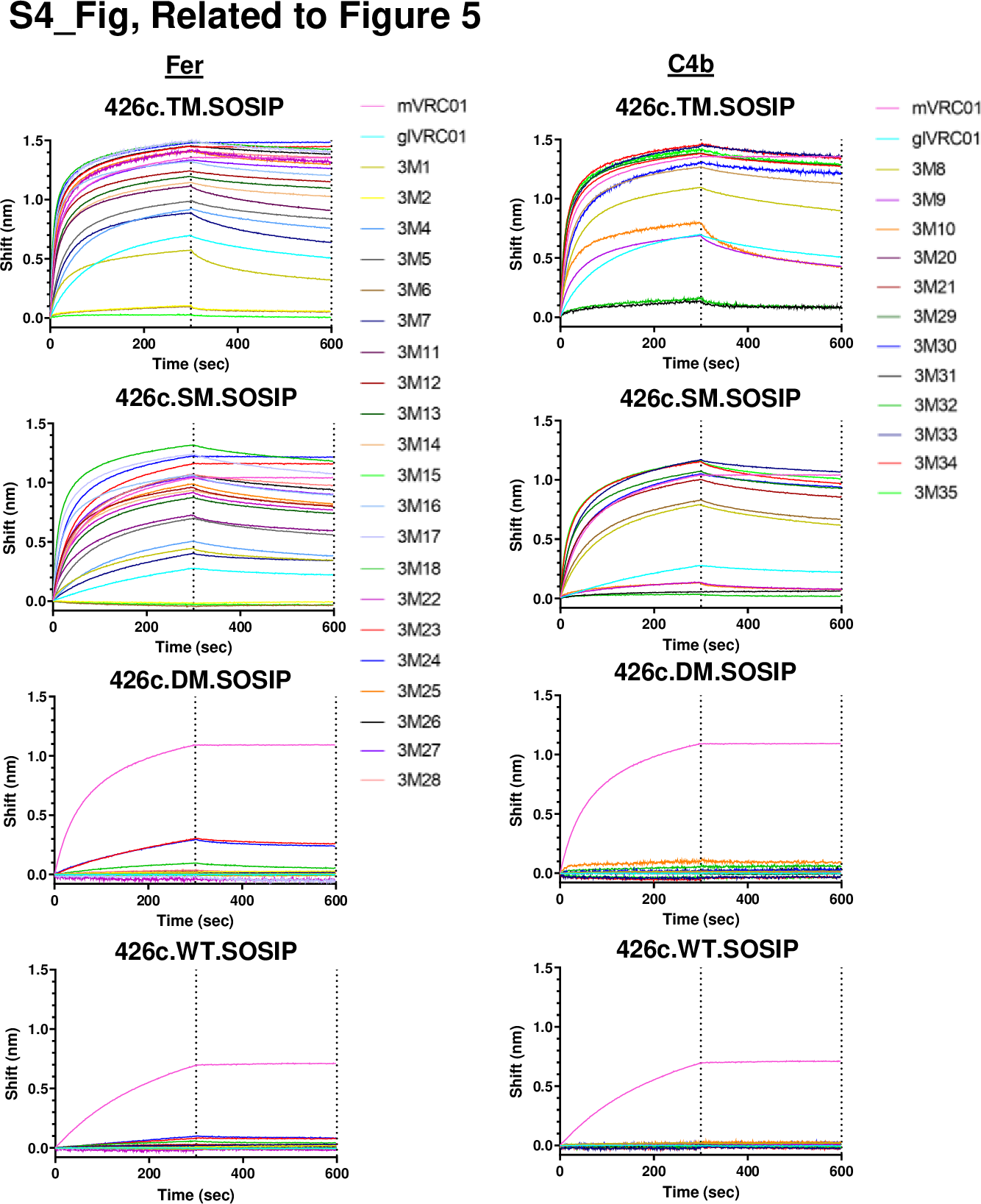

Supplement: S4 Fig — mVRC01 (solid pink line) and glVRC01 (solid cyan line) were included as internal controls. Black dotted lines indicate end of association and dissociation phases. (TIF) [file ppat.1013039.s004.tif]

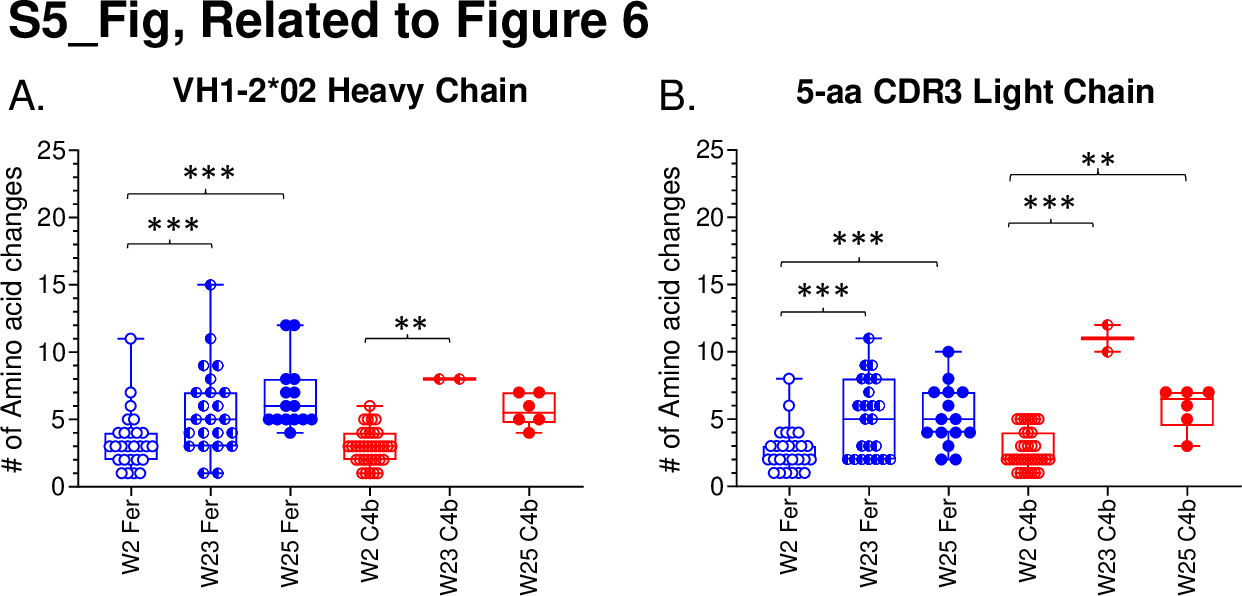

Supplement: S5 Fig — Each circle represents a paired sequence and ‘*’ indicates significant differences using Kruskal-Wallis test. (TIF) [file ppat.1013039.s005.tif]

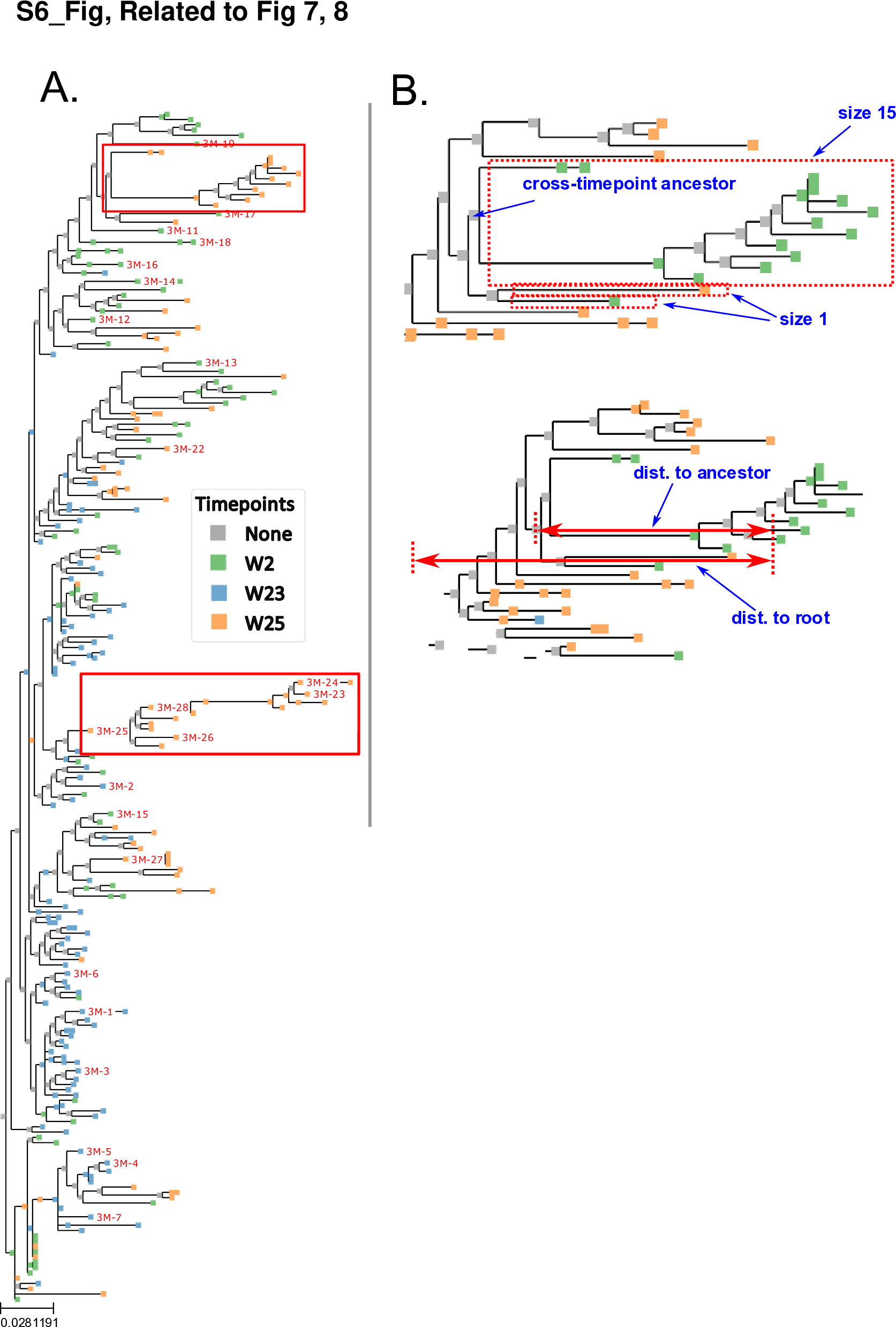

Supplement: S6 Fig — Timepoints are colored as indicated (with inferred ancestral sequences in grey), and antibodies chosen for synthesis are labeled in red. The two largest single-timepoint subtrees (see Fig 7) are indicated with red boxes. (B) Identification of single-timepoint subtrees and calculation of the resulting subtree size (top) and ancestor distance (bottom). To identify single-timepoint subtrees, for each leaf we find the largest subtree consisting entirely of nodes from a single timepoint. For all such subtrees we measure the size (number of nodes) and distance to ancestor (mean distance from nodes to common cross-timepoint ancestor, bottom). We also considered distance to root (bottom), but determined that it had less biological relation to boosting. (TIF) [file ppat.1013039.s006.tif]

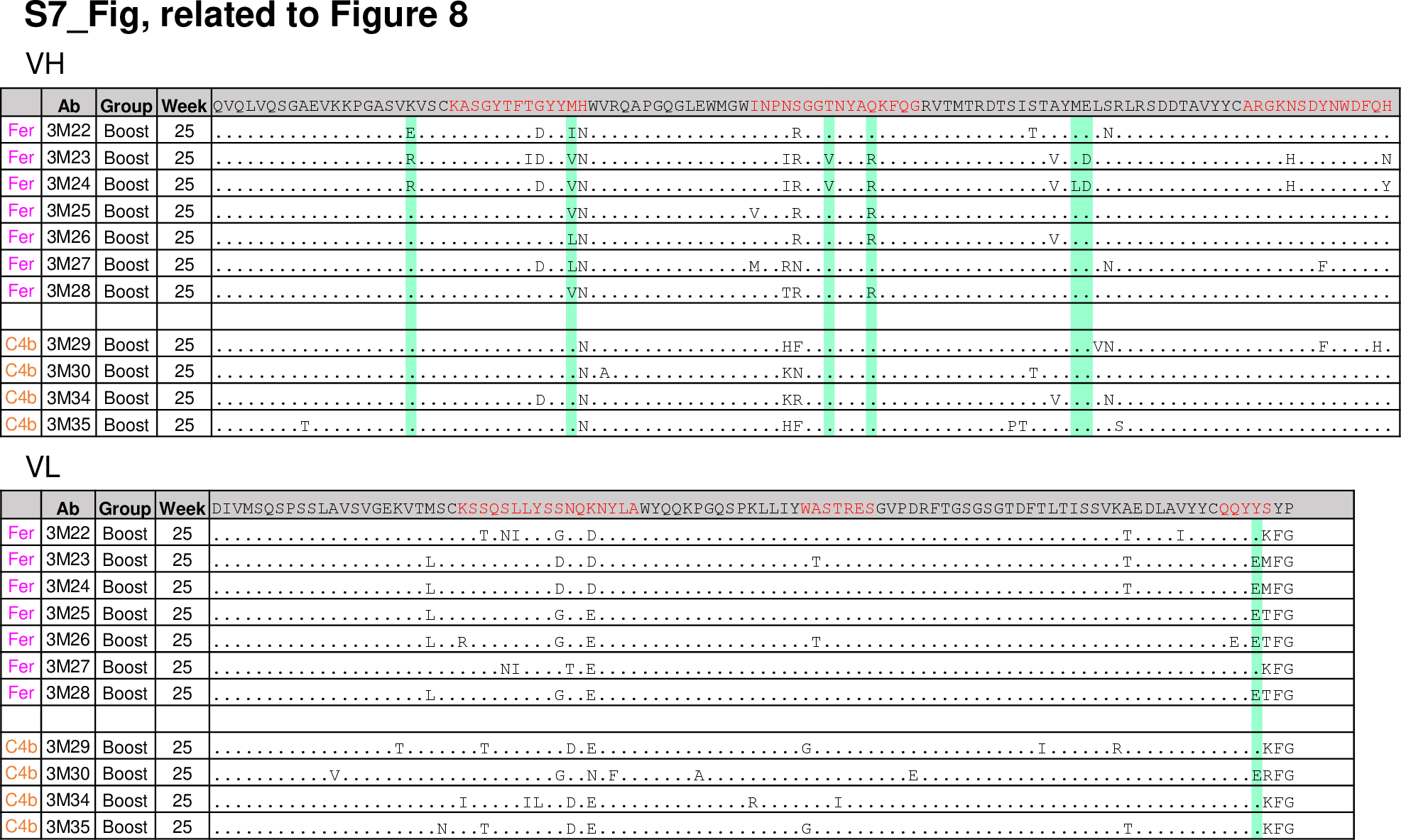

Supplement: S7 Fig — Germline VH1–2*02 and κ8–30*01 sequences are used as reference for alignment, and CDRs are highlighted in red. Green shaded regions highlight the residues commonly present in mature VRC01-class antibodies that are only, or more frequently, found in post-boost mAbs from the Fer group only. (TIF) [file ppat.1013039.s007.tif]

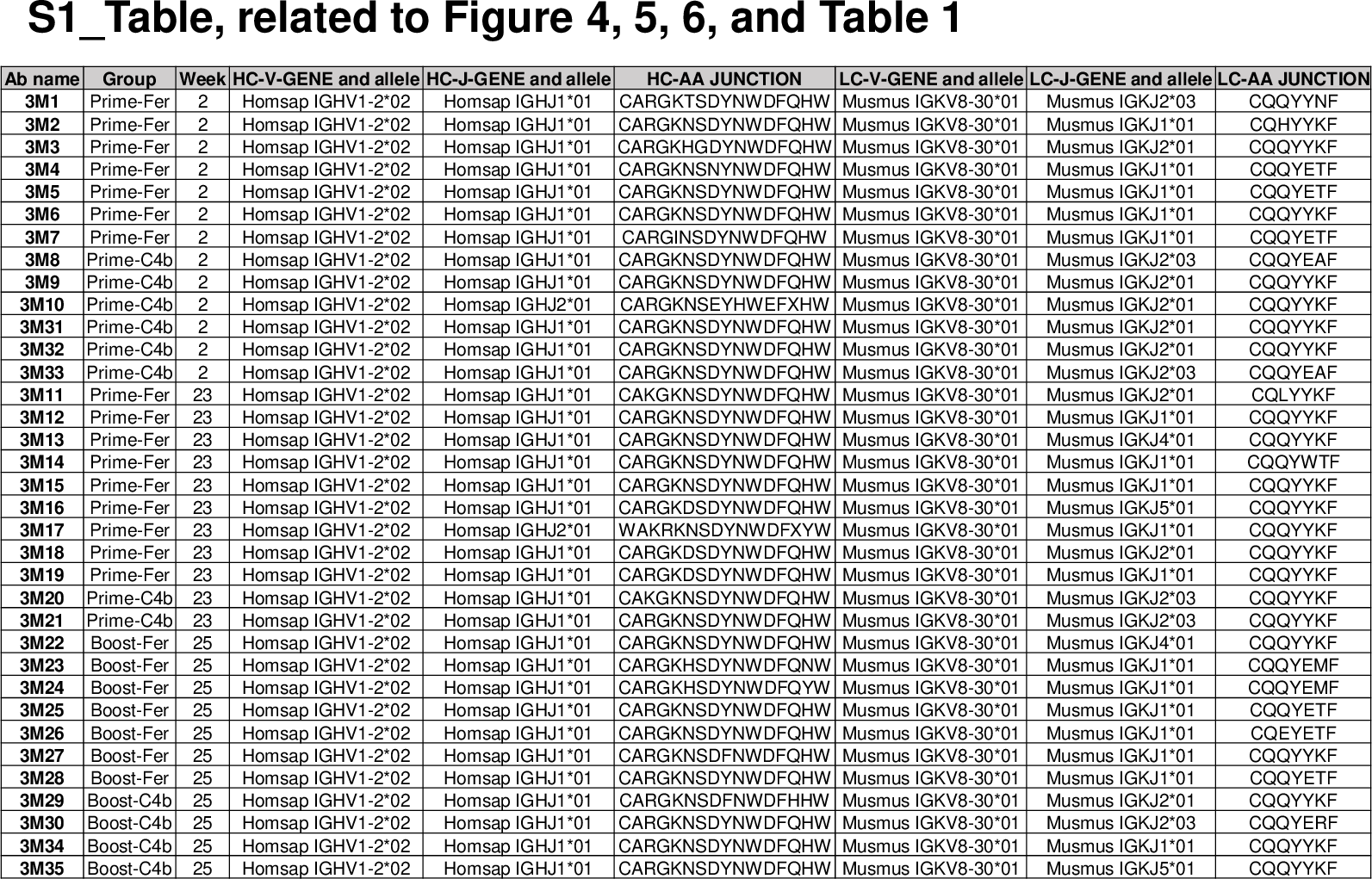

Supplement: S1 Table — A total of 33 VRC01-like mAbs were successfully generated from the immunized animals. (TIF) [file ppat.1013039.s008.tif]
